# Compassion from Others and Social Psychiatry: The Impact of Religious and Secular Frameworks of Interpretation

**DOI:** 10.1192/j.eurpsy.2026.11308

**Published:** 2026-07-31

**Authors:** C. Lazzari, P. Crawford, Y. Kotera

**Affiliations:** 1 Psychiatry, International Centre for Healthcare and Medical Education (ICHME), London; 2 Health Sciences, University of Nottingham, Nottingham, United Kingdom; 3 Center for Infectious Disease Education and Research, University of Osaka, Osaka, Japan; 4 Department of Social Sciences, Azerbaijan University, Azerbaijan, Azerbaijan

## Abstract

**Introduction:**

Compassion plays a vital role in psychological well-being and social relationships, yet little is known about how people interpret compassion from others. This study examines the ethical frameworks—both religious and secular—that influence these perceptions, providing insight into the meanings assigned to compassion in various social contexts.

**Objectives:**

The study aims to understand how working-age adults perceive compassion from others and to see how religious and secular beliefs shape these views. It also seeks to evaluate the importance of compassion in areas like healthcare, education, and media, and to identify the ethical principles that influence expectations and understanding of empathetic behavior in society.

**Methods:**

A total of 318 U.S. adults aged 18–65 participated in the study, including both genders. Participants were recruited through anonymous online opportunistic sampling, which allowed for a broad demographic representation and diverse perspectives. Chi-square Goodness of Fit Index (GFI) statistics and Cohen’s *d* effect size were used to analyze the results.

**Results:**

Respondents expressed diverse opinions on the visibility and role of compassion in society. Just over half (50.63%) felt that compassion should receive more media attention, while 37.74% believed it is adequately represented, indicating a significant difference (GFI: *p* < 0.01; *d* = 1.09). Healthcare (73.90%) and education (66.98%) were most often identified as areas needing greater compassion, followed by family, community services, and politics (GFI: *p* < 0.001; *d* = 0.34). Regarding educational integration, 71.38% supported teaching compassion in schools, with smaller groups preferring its inclusion in ethics/philosophy (11.95%) or religious studies (4.09%) (GFI: *p* < 0.001; *d* > 1.0). Daily experiences with compassion varied: 34.28% reported encountering acts of compassion from others very often, 44.65% occasionally, and only 1.57% said they never experienced it (GFI: *p* < 0.001; *d* = 1.4). When it came to understanding and guidance on compassion, opinions were divided: 36.79% regarded religion and common sense as equally influential, while nearly equal parts attributed it either to religion (28.93%) or to common sense (28.62%) alone (GFI: *p* < 0.01; *d* = 1.6).

**Conclusions:**

The findings highlight the crucial role that compassion plays in both institutional and interpersonal settings, particularly in healthcare, education, and the media. Participants’ interpretations stress the importance of both religious and secular frameworks in shaping how compassion is perceived and understood. These results highlight that any effort to foster a compassionate culture—whether through policy, teaching methods, or public messaging—must account for the complex ways belief systems influence and mediate compassionate experiences

**Disclosure of Interest:**

None Declared

